# An Efficient Microwave-Assisted Suzuki Reaction using a New Pyridine-Pyrazole/Pd(II) Species as Catalyst in Aqueous Media

**DOI:** 10.3390/molecules18021602

**Published:** 2013-01-25

**Authors:** Liqun Shen, Suyu Huang, Yuanmei Nie, Fuhou Lei

**Affiliations:** Key Laboratory of Development and Application of Forest Chemicals of Guangxi, College of Chemistry and Chemical Engineering, Guangxi University for Nationalities, Nanning, 530006, China

**Keywords:** Suzuki coupling, pyridine-pyrazole, palladium, aqueous media

## Abstract

A new pyridine-pyrazole N–N ligand has been conveniently synthesized and characterized by ^1^H-, ^13^C-NMR, IR spectroscopies, HRMS and X-ray single-crystal crystallography analyses. The ligand adds to palladium(II) under basic conditions to give high yields of an air-stable and water-soluble complex that was fully characterized by NMR and HRMS. The complex was investigated as a catalyst for the Suzuki reaction in aqueous media under microwave irradiation. The compound proved to be an effective catalyst.

## 1. Introduction

In the past three decades, the palladium-catalyzed Suzuki reaction of aryl halides with aryl boronic acids has been one of the most important and efficient methods for the formation of unsymmetrical biaryl compounds [1,2], which are extensively found in a range of natural products [3], pharmaceuticals [4], ligands [5], herbicides and advanced materials [6]. Modern techniques have been developed in order to enable simpler, faster and cheaper versions of already known chemical transformations to meet the purposes of Green Chemistry [7]. Recently, the use of aqueous phases including water and water/organic mixtures as solvents for the Suzuki reaction has also received considerable attention, as water is cheap, environmental friendly, inflammable, and allows simple separation and catalyst recycling [8,9]. For these reasons, much effort has been directed to perform the Suzuki coupling in neat water. Casalnuovo [10] was the first to demonstrate that the palladium-catalyzed cross-coupling reactions could be carried out in aqueous solvents catalyzed by TPPMS/Pd(OAc)_2_, and since then, other hydrophilic ligands for aqueous-phase Suzuki cross-coupling reactions have been developed [11]. Recently the application of nitrogen-based ligands systems, such as Schiff bases, guanidine, aryloximes, arylimines, has also been found to produce highly active catalysts for Suzuki reaction in water [12,13,14,15,16,17]. Nowadays, microwave heating has been widely exploited in organic synthesis and its advantages over traditional heating lay in the reduction in reaction time from hours to minutes [18,19]. In addition, microwave heating is generally able to reduce side reactions, increase yields and improve reproducibility [20]. Microwave-assisted Suzuki reactions can be considered today as an efficient synthetic methodology [21,22,23,24,25].

To the best of our knowledge, Pd(II) complexes with the pyridine-pyrazole ligand have been examined for cytotoxic activity and few investigations have been carried out on their catalytic activity [26,27,28]. In this paper, we report carboxylated water-soluble pyridine-pyrazole ligands as supporting ligands for the Suzuki reaction in water and in aqueous phases in conjunction with microwave heating.

## 2. Results and Discussion

### 2.1. Synthesis and Characterization of the Ligand and Complee

**Scheme 1:**
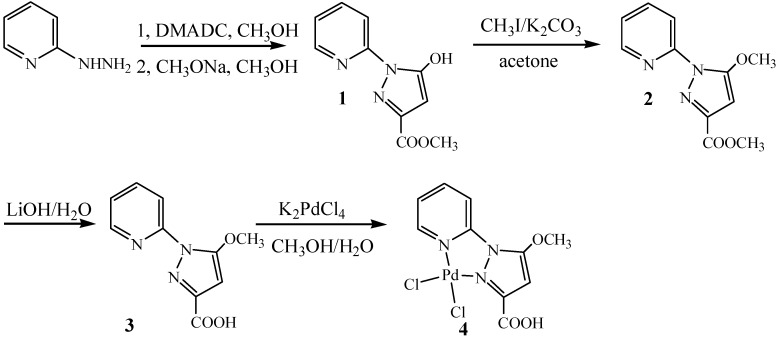
Synthesis of pyridine-pyrazole ligand **3** and the Pd(II) complex **4**.

The synthetic pathway for compounds **3** and **4** was shown in Scheme 1. 5-Hydroxy-1-pyridin-2-yl-1*H*-pyrazole-3-carboxylic acid methyl ester (**1**) was easily synthesized by the reaction of 2-hydrazinopyridine and dimethyl acetylenedicarboxylate (DMADC), with subsequent cyclization in methanolic NaOCH_3_ solution.

**Figure 1:**
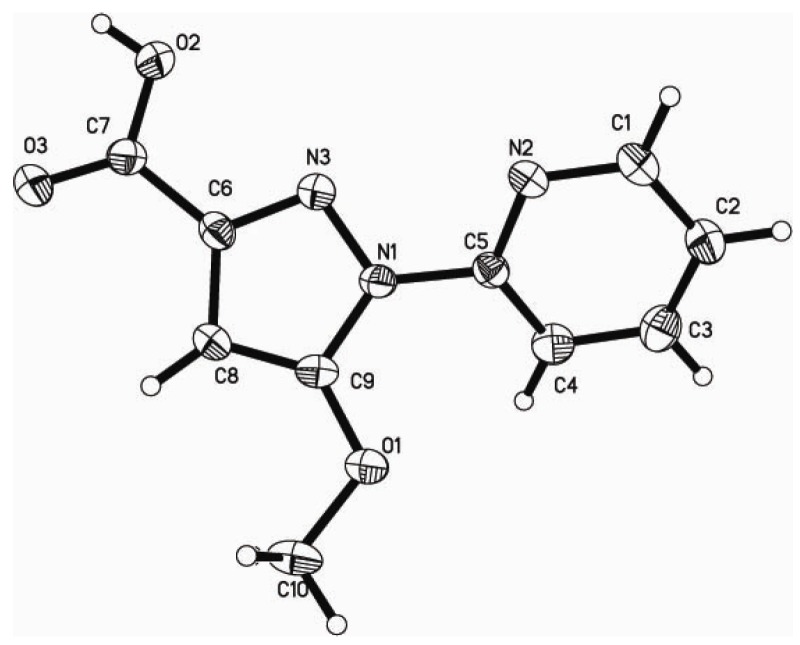
The molecular structure of the compound **3** with atom-numbering scheme, displacement ellipsoids are drawn at the 30% probability level.

**Figure 2:**
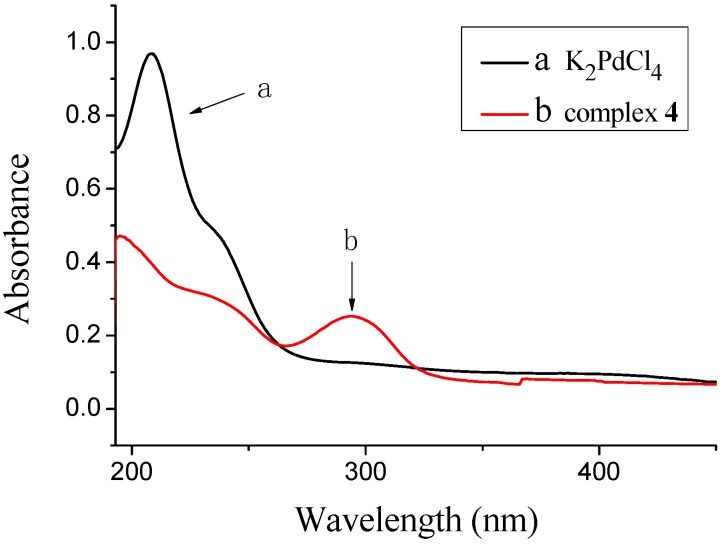
(**a**) UV-vis spectra of K_2_PdCl_4_ in water; (**b**) UV-vis spectra of complex **4** in water.

The alkylation of **2** with iodomethane provided 5-methoxy-1-pyridin-2-yl-1*H*-pyrazole-3-carboxylic acid methyl ester (**2**). Compound **2** was hydrolyzed with LiOH in methanol at room temperature to afford 5-methoxy-1-pyridin-2-yl-1*H*-pyrazole-3-carboxylic acid (**3**) bearing a carboxylic acid group. Compound **3** has been characterized by ^1^H-NMR, ^13^C-NMR, IR and high resolution mass spectrometry (HRMS). The solid-state structure has been established by X-ray single-crystal crystallography [29] (Figure 1). The reaction of a methanol solution of ligand **3** with an equimolar amount of an aqueous solution of K_2_PdCl_4_ produced the palladium(II) complex (**4**) in 81% yield as yellow crystals. The mononuclear complex with a pyridine-pyrazole *N*,*N*-chelate has been characterized by IR, UV/vis (Figure 2), NMR and high resolution mass spectrometry.

We now observe the interaction of K_2_PdCl_4_ with the ligand **3** by UV/vis spectroscopy. In Figure 2, as can be seen, the spectrum of K_2_PdCl_4_ displays one major absorption band at 208 nm, that changes upon addition of ligand **3** (195 and 294 nm). This change in the UV/vis spectrum can be attributed to the coordination of Pd(II) to the ligand **3** [30].

### 2.2. Catalysis of the Suzuki Reaction

As a starting point for the development of our microwave-promoted methodology, the Suzuki reaction were performed using a scientific microwave apparatus [31], working on a 1 mmol scale in a 10 mL sealed glass vessel. The Suzuki reaction of different types of aryl halides using complex **4** as catalyst was then investigated under microwave irradiation. Initially, in order to optimize the reaction conditions, we employed the coupling reaction of 4'-bromoacetophenone with phenylboronic acid in water/ethanol as a model reaction to investigate the effect of different bases on the reaction. Water/ethanol as solvent facilitates solvation of the aryl halide in neat water [32]. For this purpose, this reaction was performed using different bases in the presence of 0.1 mol% of complex **4** as catalyst. Using a microwave power of 60 W, we ramped the temperature from 25 °C to 120 °C, and then held it at 120 °C for 2 min. The results are summarized in Table 1. The best results were obtained using KOH as the base (Table 1, entries 1–4), other bases, such as K_3_PO_4_, K_2_CO_3_ and Et_3_N were slightly less efficient. We next investigated the effect of different solvents for the same reaction (Table 1, 5–8). As can be seen in Table 1, the best result was obtained using aqueous EtOH/H_2_O as the solvent (Table 1), perhaps, attributable to the better solubility of the reagents and easier reduction of Pd^2+^ to Pd(0).

**Table 1:** Optimization of base and solvent for Suzuki cross-coupling reaction under microwave irradiation ^a^.

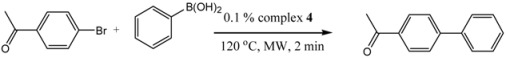			
Entry	Base	Solvent	Conversion (%) ^b^
1	KOH	EtOH/H_2_O	99
2	K_3_PO_4_	EtOH/H_2_O	95.2
3	K_2_CO_3_	EtOH/H_2_O	96.0
4	Et_3_N	EtOH/H_2_O	92.3
5	KOH	DMF/H_2_O	90.9
6	KOH	DMAC/H_2_O	85.4
7	KOH	MeCN/H_2_O	94.5
8	KOH	H_2_O	85.0

^a^ *Reaction conditions*: 1 mmol 4'-bromoacetophenone, 1.3 mmol phenylboronic, 2 mmol base, 2 mL solvent (EtOH/H_2_O = 1:1; MeCN/H_2_O = 1:1; DMF/H_2_O = 1:1; DMA/H_2_O = 1:1), Microwave irradiation = 60 W. and 0.1 mol% complex **4**. DMF = *N*,*N*-Dimethylformamide; DMAC = *N*,*N*-Dimethylacetamide; ^b^ Determined by HPLC analysis.

Under the above optimized conditions, complex **4** was applicable to a wide range of aryl bromides, iodides, and less reactive aryl chlorides and the results are summarized in Table 2. Generally, the catalyst was very effective when electron poor or electron-rich aryl bromides were used (Table 2, entry 1–7). From Table 2, it can be seen that the electronic and steric characters of the aryl bromides have an effect on the Suzuki reactions under the optimized conditions. For this purpose we applied 2-, 3-, and 4-bromobenzaldehyde under the optimized reaction conditions (Table 2, entries 1–3). The Suzuki reaction of 4-bromobenzaldehyde was complete and gave excellent yields. Sterically demanding 2-bromobenzaldehyde resulted in decreased coupling. The reaction of 4-chlorobromobenzene with phenylboronic acid produced mono-substituted 4-chlorobiphenyl (Table 2, entry 8), the result indicates that the catalytic reaction has a good chemical selectivity. The coupling reaction using 4-methoxyphenylboronic acid was also performed, the corresponding products are obtained in good yields (Table 2, entry 14–26). We also investigated the scope of this method on aryl chlorides, and found that the conversion of activated aryl chloride was up to 89.0% (Table 2, entry 26) and unactivated aryl chlorides gave moderate yields.

**Table 2:** Suzuki coupling of aryl halides and aryl boronic acids in H_2_O/EtOH using complex **4** under optimized reaction conditions under microwave irradiation ^a^.

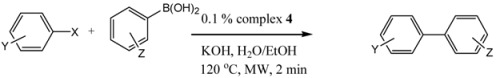				
Entry	X	Y	Z	Yield (%) ^b^
1	Br	4-CHO	H	90.3
2	Br	3-CHO	H	85.1
3	Br	2-CHO	H	80.3
4	Br	4-OMe	H	86.7
5	Br	4-CH_3_	H	82.4
6	Br	4-OH	H	93
7	Br	4-COMe	H	92.6
8	Br	4-Cl	H	90.8
9	Br	H	H	86.7
10	I	H	H	91.3
11	Cl	H	H	63.8
12	Cl	2-COOH	H	75.5
13	Cl	4-NO_2_	H	88.3
14	Br	4-CHO	4-OMe	92.7
15	Br	3-CHO	4-OMe	87.7
16	Br	2-CHO	4-OMe	83.5
17	Br	4-OMe	4-OMe	88.1
18	Br	4-CH_3_	4-OMe	85.3
19	Br	4-OH	4-OMe	93.2
20	Br	4-COMe	4-OMe	93.0
21	Br	4-Cl	4-OMe	91.2
22	Br	H	4-OMe	90.8
23	I	H	4-OMe	92.7
24	Cl	H	4-OMe	67.2
25	Cl	2-COOH	4-OMe	78.4
26	Cl	4-NO_2_	4-OMe	89.0

^a^ *Reaction conditions*: 1 mmol 4'-bromoacetophenone, 1.3 mmol phenylboronic, 2 mmol KOH, 2 mL solvent (EtOH/H_2_O = 1:1), Microwave irradiation = 60 W, and 0.1 mol% complex **4**; ^b^ Isolated yield after purification by column chromatography.

### 2.3. Catalyst Recycling

The potential recyclability of the catalysts derived from the pyridine-pyrazole/Pd system was examined using the model cross-coupling of 4-bromoacetophenone with phenylboronic acid. The reaction was carried out in 1:1 H_2_O/CH_3_CH_2_OH under microwave irradiation for 2 min. After extracting the products with dichloromethane the yields were determined by HPLC. The resulting aqueous solution was recharged with the same substrates for the next cycle. It was shown that the catalytic solution could be reused for third cycles, and by the fifth cycle, the yield had dropped to 21% (Table 3).

**Table 3:** Recycling of catalyst ^a^.

Cycles(n)	1	2	3	4	5
Conversion (%) ^b^	99	97	86	53	21

^a^ *Reaction conditions*: 1 mmol 4'-bromoacetophenone, 1.3 mmol phenylboronic, 2 mmol KOH, 120 °C under microwave irradiation 2 min; ^b^ Determined by HPLC analysis.

## 3. Experimental

### 3.1. General

All chemicals were of reagent grade and used as commercially purchased without further purification. All solvents were purified according to standard procedures. Melting points were determined using a WRS-1B apparatus and were uncorrected. The IR spectra were recorded in the range of 400–4000 cm^−1^ with a Magna 550 FT-IR spectrometer using KBr pellets. The ^1^H and ^13^C-NMR spectra were recorded on a Bruker AV600 spectrometer. High resolution mass (HRMS) spectra were obtained in ESI mode on a Finnigan MAT95XP HRMS system (Thermo Electron Corporation). HPLC analysis of the target compound was performed on a C18 reversed-phase column (5 µm, 200 × 4.6 mm) using water-methanol (20:80, v/v) as the mobile phase at a flow rate of 0.8 mL/min. Microwave reactions were carried out in a Biotage Initiator 60.

### 3.2. Synthesis and Characterization

*5-Hydroxy-1-pyridin-2-yl-1H-pyrazole-3-carboxylic acid methyl ester* (**1**) [33]. Dimethyl acetylenedicarboxylate (7.5 mL, 61 mmoL) in methanol (15 mL) was added dropwise to a solution of 2-pyridylhydrazine (6.0 g, 55 mmoL) in methanol (60 mL), and the mixture was stirred for 6.0 h at 0 °C. Sodium methoxide (4.6 g, 85.1 mmoL) was then added and stirring was continued for 30 min at 70 °C. After cooling, and quenched with water. The reaction mixture was filtered and washed with cold methanol, and dried in vacuo to give a white solid. Yield: 8.9 g (77.4%). M.p.: 142.2–143.0 °C; ^1^H-NMR (600 MHz, CDCl_3_) δ 3.95 (s, 3H), 6.10 (s, 1H), 7.27–7.31 (m, 1H), 7.91–7.98 (m, 1H), 8.11 (dd, *J* = 5.0, 4.2 Hz, 1H), 8.31–8.36 (m, 1H), 12.83(s, 1H). ppm. ^13^C-NMR (151 MHz, CDCl_3_) δ 52.4, 90.3, 111.3, 121.6, 140.4, 144.5, 145.4, 154.2, 157.1, 162.8 ppm; HRMS (M+H^+^) calcd. for C_10_H_10_N_3_O_3_ 220.0722, found 220.0726. IR (KBr): 3471, 3030, 2957, 1744, 1612, 1588, 1452, 1254, 775 cm^−1^.

*5-Methoxy-1-pyridin-2-yl-1H-pyrazole-3-carboxylic acid methyl ester* (**2**). Potassium carbonate (1.23 g, 9.91 mmoL) was added to stirred solution of 1-(pyridin-2-yl)-5-hydroxy-1*H*-pyrazole-3-carboxylicacid methyl ester (**1**, 0.65 g, 2.97 mmoL) in acetone (25 mL) at 50 °C, methyl iodide (0.4 mL) was added under argon. The mixture allowed to be stirred at 50 °C for 12 h, the reaction mixture was filtered and extracted with CHCl_3_ three times, after evaporating the organic layers under pressure, the residue was purified by chromatography on silica gel to give a pale white solid. Yield: 0.53 g (76.6%). M.p.: 108.7–116.6 °C; ^1^H-NMR (600 MHz, CDCl_3_) δ 3.94 (s, 3H), 4.00 (s, 3H), 6.26 (s, 1H), 7.28–7.31 (m, 1H), 7.75–7.77 (m, 1H), 7.83–7.84 (m, 1H), 8.53–8.59 (m, 1H) ppm. ^13^C-NMR (151 MHz, CDCl_3_) δ 52.2, 59.7, 88.5, 117.8, 122.8, 138.5, 143.5, 148.7, 151.0, 156.8, 162.8 ppm; HRMS (M+H^+^) calcd. for C_11_H_12_N_3_O_3_ 234.0878, found 234.0880. IR (KBr): 3159, 3086, 2947, 1737, 1595, 1564, 1480, 1473, 1230, 1067, 754 cm^−1^.

*5-Methoxy-1-pyridin-2-yl-1H-pyrazole-3-carboxylic acid* (**3**). Lithium hydroxide (190 mg, 4.5 mmoL) was added to a solution of 1-(pyridin-2-yl)-5-methyl ether-1*H-*pyrazole -3-carboxylic acid methyl ester (**2**, 0.35 g, 1.5 mmoL) in methanol (18 mL) and water (3 mL),The mixture was stirred at room temperature for 12 h. After completion of the reaction, extracted with chloroform (3 × 15 mL), and evaporating the organic layers under reduced pressure, the residue recrystallised from absolute ethyl alcohol to obtain the white product. Yield: 0.24 g (73%). M.p.: 201.1–203.5 °C; ^1^H-NMR (600 MHz, DMSO) δ 3.96 (s, 3H), 6.33 (s, 1H), 7.46 (ddd, *J* = 7.5, 4.9, 0.6 Hz, 1H), 7.67 (d, *J* = 8.1 Hz, 1H), 8.02 (td, *J* = 7.8, 1.9 Hz, 1H), 8.55 (dd, *J* = 4.8, 1.6 Hz, 1H), 12.93 (s, 1H) ppm. ^13^C-NMR (151 MHz, DMSO) δ 59.6, 88.4, 118.0, 123.4, 139.1, 143.6, 148.6, 150.3, 156.3, 163.0 ppm; HRMS (M+H^+^) calcd. for C_10_H_10_N_3_O_3_ 220.0722, found 220.0726. IR (KBr): 3444, 3134, 3114, 2997, 1701, 1604, 1574, 1484, 1439, 1242, 793 cm^−1^.

*Palladium(II) complex* **4**. An aqueous solution (4 mL) of K_2_[PdCl_4_] (66.3 mg, 0.2 mmoL) was added dropwise to a stirred methanol solution (20 mL) of ligand **3** (43.7 g, 0.2 mmol) at room temperature [34]. During the addition a solid product precipitated immediately from the reaction mixture. Stirring was continued for 30 min. The stable yellow complex was filtered off, washed with cold water, and dried; 68.3 mg yield (83.3%), m.p. > 300 °C; ^1^H-NMR (600 MHz, DMSO) δ 3.95 (s, 3H), 6.32 (s, 1H), 7.47 (m, 1H), 7.67 (d, *J* = 8.2 Hz, 1H), 8.00 (m, 1H), 8.55 (d, *J* = 4.9 Hz, 1H), 12.93 (s, 1H) ppm. ^13^C-NMR (151 MHz, DMSO) δ 59.5, 88.4, 118.2, 123.4, 139.2, 143.6, 148.7, 150.2, 156.4, 163.1 ppm; HRMS (M+H^+^) calcd. for C_10_H_10_C_l2_N_3_O_3_Pd 395.9056, found 395.9055. IR (KBr): 3430, 3110, 2936, 1664, 1609, 1584, 1511, 1487, 1317, 771, 469 cm^−1^.

### 3.3. Crystallography

A suitable crystal was obtained from an ethanol solution by slow evaporation. Diffraction experiments for **3** was carried out on with *Mo K_a _*radiation (*λ* = 0.71073 Å) using a Bruker SMART APEX CCD diffractometer at 296 K. Raw frame data were integrated with the SAINT program. The structures were solved by direct methods which gave the positions of all non-hydrogen atoms and refined with full-matrix least-squares on *F*^2^ using SHELXS-97 and SHELXL-97 [35]. The hydrogen atoms were set in the calculated positions and refined by riding model. The crystallographic and refinement data of **3** is listed in Table 4.

### 3.4.General Procedure for Aqueous Suzuki Coupling Reactions

Into a 10 mL glass vial were placed aryl halide (1.0 mmol), phenylboronic acid (1.2 mmol), base (2 mmol), complex **4** (0.4 mg, 0.001 mmol), ethanol (1 mL), water (1 mL), and a magnetic stir bar. The vessel was sealed by capping with a Teflon septum fitted in an aluminum crimp top and placed into the microwave cavity. Microwave irradiation of 60 W was used, the temperature being ramped from room temperature to 120 °C. Once 120 °C was reached, the reaction mixture was held at this temperature for 2 min. After the mixture was allowed to cool to room temperature, the reaction vessel was opened and the contents poured into a separating funnel. Water and dichloromethane (30 mL of each) were added, and the organic material was extracted and removed. After further extraction of the aqueous layer with dichloromethane, combining of the organic washings and drying over MgSO_4_, the dichloromethane was removed *in vacuo*, leaving the crude product. The crude material was flash chromatographed on a silica gel column. All of the compounds have been characterized by comparing ^1^H-NMR with the values found in the literature [35,36,37,38,39,40,41].

**Table 4:** Crystal data and refinement details for **3**.

Compound reference	3
Empirical formula	C_10_H_5_N_3_O_3_
Formula weight	215.17
Crystal system	Monoclinic
Space group	*P2(1)/n*
Unit cell dimensions	*a* =7.144(3) Å, *α* =90.00;
	*b* = 8.093(4)Å, *β* = 95.6380(10);
	*c* = 16.662(3)Å, *γ* = 90.00
Volume	958.6(6)Å^3^
Z	4
Density(calculated)	1.491 g/cm^3^
Absorption coefficient	0.115 mm^−1^
*F(000)*	440
Crystal size	0.27 × 0.25 × 0.22 mm^3^
Theta range for data collection (°)	2.46–25.00 °
Reflections collected	4745
Completeness to θmax	0.993
Data/restraints/parameters	1678/0/146
Goodness-of-fit on *F^2^*	0.917
Final R indices [I > 2σ(I)] ^a,b^	R_1_ = 0.0612， *w*R_2_ = 0.1673
R indices (all data)	R_1_ = 0.0940， *w*R_2_ = 0.2057
Largest diff. peak and hole	0.309 and −0.294e Å^−3^

^a^ *R*_1_ = ∑||*F*_o_|-|*F*_c_||/∑|*F*_o_|; ^b^ *wR*_2_ = [∑*w*(*F*_o_^2^-*F*_c_^2^)^2^/∑*w*(*F*_o_^2^)^2^]^1/2^, *w* = [^2^(*F*_o_)^2^ + (0.1(max(0, *F*_o_^2^) + 2*F*_c_^2^)/3)^2^]^−1^.

### 3.5. Recycling of Catalyst

Into a 10 mL glass vial were placed 4-bromoacetophenone (1.0 mmol), phenylboronic acid (1.2 mmol), KOH (2 mmol), complex **4** (0.4 mg, 0.001 mmol), ethanol (1 mL), water (1 mL), and a magnetic stir bar. The reaction mixtures were run at 120 °C for 2 min under microwave irradiation. After the mixture was allowed to cool to room temperature, the reaction vessel was opened and the contents poured into a separating funnel. Diethyl ether (5 mL × 2) was added. The aqueous phase was transferred to another glass vial for the next reaction cycle. The yields were determined by HPLC.

## 4. Conclusions

In summary, a new and efficient catalyst system for microwave-mediated Suzuki coupling reaction was developed. To the best of our knowledge this is the first report of pyridine-pyrazole/Pd(II) complexes for catalysis of Suzuki reaction. The reactions were performed under microwave irradiation in water/EtOH co-solvent system and have shown the application of this to the synthesis of a number of biaryls. The method has the advantage of rapid reaction times, no need for anaerobic conditions and use of a nontoxic solvent. The catalysts could be used for up to five cycles. The methodology is a good synthetic route for biaryl synthesis.

## Supplementary Materials

Supplementary materials can be accessed at: http://www.mdpi.com/1420-3049/18/2/1602/s1.
