# Supplementary material for: An Efficient Microwave-Assisted Suzuki Reaction using a New Pyridine-Pyrazole/Pd(II) Species as Catalyst in Aqueous Media
Source: Molecules. 2013 Jan 25;18(2):1602–12. doi: 10.3390/molecules18021602 (PMC6270469; doi:10.3390/molecules18021602)
Supplement: Supplementary file 1 [file molecules-18-01602-s001.pdf]

Supplementary Information

^{1}H-NMR of the coupling products

4-Biphenylcarbaldehyde (Table 2, entry 1)

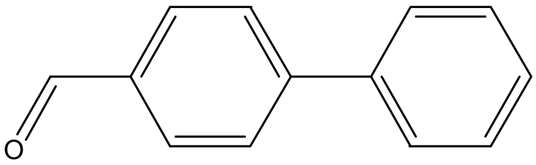

^{1}H-NMR (600 MHz, $CDCl_3$) δ 10.06 (s, 1H), 7.97–7.5 (m, 2H), 7.77–7.75 (m, 2H), 7.64 (ddd, *J* = 4.1, 3.2, 1.8 Hz, 2H), 7.50–7.47 (m, 2H), 7.43–7.41(m, 1H).

Biphenyl-3-carbaldehyde (Table 2, entry 2)

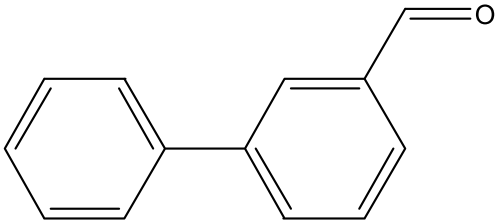

^{1}H-NMR (600 MHz, $CDCl_3$) δ 10.09 (s, 1H), 8.11 (t, *J* = 1.7 Hz, 1H), 7.86 (dd, *J* = 7.6, 1.7 Hz, 2H), 7.64–7.53 (m, 3H), 7.50–7.46 (m, 2H), 7.40–7.39 (m, 1H).

Biphenyl-2-carbaldehyde (Table 2, entry 3)

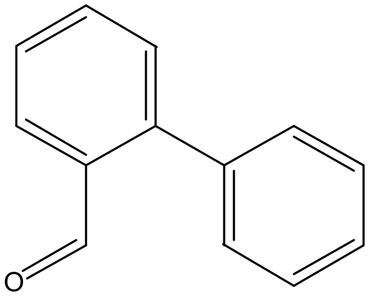

^{1}H-NMR (600 MHz, $CDCl_3$) δ 10.00 (s, 1H), 8.05–8.03 (m, 1H), 7.63 (td, *J* = 7.5, 1.4 Hz, 1H), 7.50–7.42 (m, 5H), 7.39–7.37 (m, 2H).

4- Methoxybiphenyl (Table 2, entry 4)

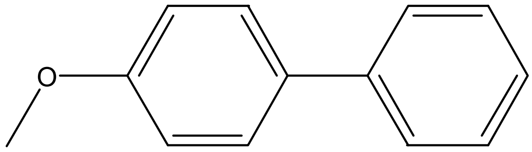

^{1}H-NMR (600 MHz, $CDCl_3$) δ 7.56–7.52 (m, 4H), , 7.44 (t, *J* = 7.6 Hz, 2H), 7.31–7.27 (m, 1H), 7.00 (dd, *J* = 13.9, 8.6 Hz, 2H), 3.88 (s, 3H).

4-Methylbiphenyl (Table 2, entry 5)

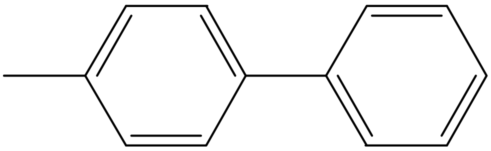

^{1}H-NMR (600 MHz, $CDCl_3$) δ 7.60 (d, *J* = 7.5 Hz, 2H), 7.50 (d, *J* = 8.1 Hz, 2H), 7.44 (d, *J* = 7.7 Hz, 2H), 7.33 ((t, *J* = 7.3 Hz, 1H), 7.26 (d, *J* = 7.5 Hz, 2H), 2.41 (s, 3H).

4-Hydroxybiphenyl (Table 2, entry 6)

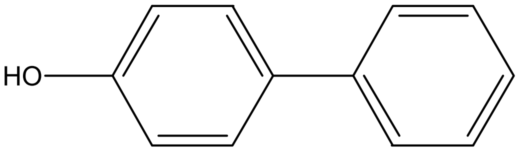

^{1}H-NMR (600 MHz, $CDCl_3$) δ 7.49 (d, *J* = 7.2 Hz, 2H), 7.37–7.28 (m, 5H), 6.72 (d, *J* = 7.8 Hz, 1H), 5.73 (s, br, 1H).

4-Acetylbiphenyl (Table 2, entry 7)

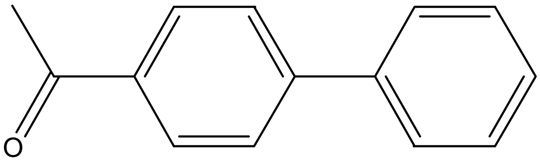

^{1}H-NMR (600 MHz, $CDCl_3$) δ 8.05–8.03 (m, 2H), 7.7–7.69 (m, 2H), 7.64–7.62 (m, 2H), 7.49–7.46 (m, 2H), 7.42–7.39 (m, 1H), 2.64 (s, 3H).

4-Chlorobiphenyl (Table 2, entry 8)

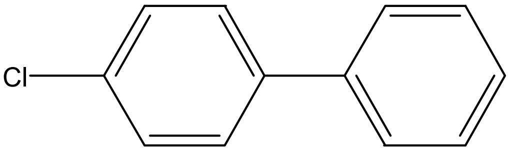

^{1}H-NMR (600 MHz, $CDCl_3$) δ 7.62–7.52 (m, 4H), 7.47–7.35 (m, 5H).

Biphenyl (Table 2, entry 9-11)

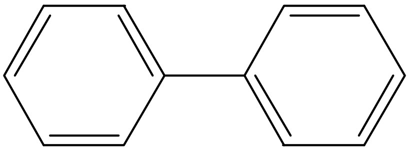

^{1}H-NMR (600 MHz, $CDCl_3$) δ 7.60 (dt, *J* = 8.1, 1.5 Hz, 4H), 7.48–7.40 (m, 4H), 7.35 (ddd, *J* = 7.0, 2.2, 1.1 Hz, 2H).

Biphenyl-2-carboxylic acid (Table 2, entry 12)

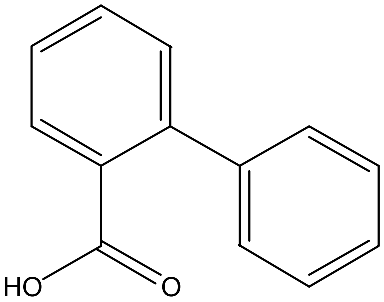

^{1}H-NMR (600 MHz, $CDCl_3$)δ 11.21 (s, 1H), 8. 05(d, J = 8.2Hz 2H), 7.74 (d, J = 8.0 Hz, 2H), 7.53–7.49 (m, 2H), 7.24–7.20 (m, 3H).

4-Nitrobiphenyl (Table 2, entry 13)

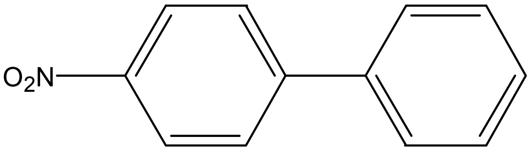

^{1}H-NMR (600 MHz, $CDCl_3$) δ 8.30 (d, J = 9.0 Hz, 2H), 7.72 (d, J = 9.0 Hz, 2H), 7.61 (d, J = 7.0 Hz, 2H), 7.48 (t, J = 7.5 Hz, 2H), 7.43 (t, J = 7.2 Hz, 1H).

4'-Methoxy-4-biphenylcarbaldehyde (Table 2, entry 14)

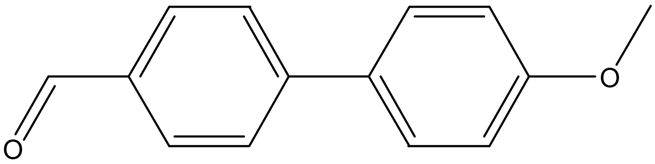

^{1}H-NMR (600 MHz, $CDCl_3$) δ 10.04 (s, 1H), 7.94–7.92 (m, 2H), 7.72 (d, J = 8.3 Hz, 2H), 7.60–7.59 (m, 2H), 7.02–7.00 (m, 2H), 3.87 (s, 3H).

4'-Methoxy-3-biphenylcarbaldehyde (Table 2, entry 15)

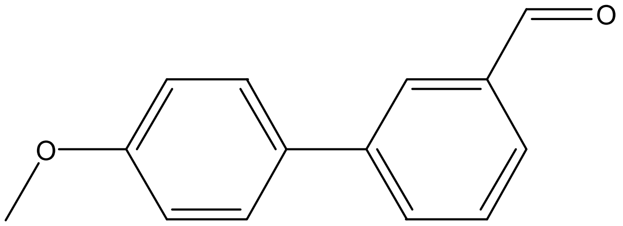

^{1}H-NMR (600 MHz, $CDCl_3$) δ 10.09 (s, 1H), 8.07 (t, J = 1.6 Hz, 1H), 7.82–7.81 (m, 2H), 7.60–7.57 (m, 3H), 7.02–7.00 (m, 2H), 3.87 (s, 3H).

4'-Methoxy-2-biphenylcarbaldehyde (Table 2, entry 16)

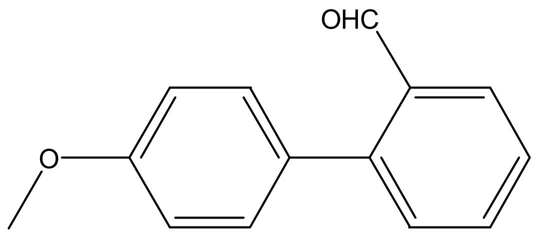

^{1}H-NMR (600 MHz, $CDCl_3$) δ 10.00 (s, 1H), 8.01 (d, *J* = 7.8 Hz, 1H), 7.62 (t, *J* = 7.5 Hz, 1H), 7.48–7.43 (m, 2H), 7.31 (d, *J* = 8.2 Hz, 2H), 7.01 (d, *J* = 8.2 Hz, 2H), 3.88 (s, 3H).

4'-Methoxy-4-methoxybiphenyl (Table 2, entry 17)

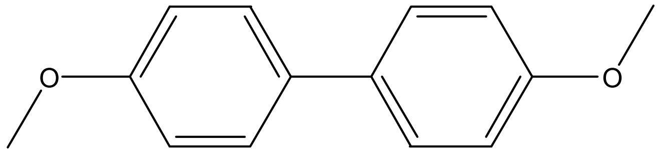

^{1}H-NMR (600 MHz, $CDCl_3$) δ 7.49–7.47 (m, 4H), 6.97–6.95 (m, 4H), 3.84 (s, 6H).

4'-Methyl -4- methoxybiphenyl (Table 2, entry 18)

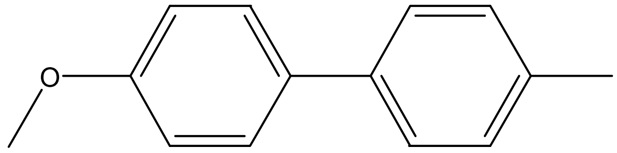

^{1}H-NMR (600 MHz, $CDCl_3$) δ 7.52–7.45 (m, 4H), 7.24–7.22 (m, 2H), 6.97 (d, *J* = 8.6 Hz, 2H), 3.85 (s, 3H), 2.39 (s, 3H).

4'-Methoxy-4-hydroxybiphenyl (Table 2, entry 19)

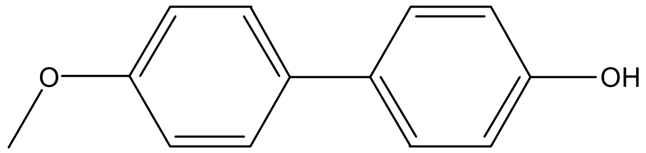

^{1}H-NMR (600 MHz, $CDCl_3$) δ 7.44 (dd, *J* = 22.1, 8.4 Hz, 4H), 6.96 (d, *J* = 8.5 Hz, 2H), 6.88 (d, *J* = 8.4 Hz, 2H), 4.78 (s, 1H), 3.84 (s, 3H).

4'-Methoxy-4-acetylbiphenyl (Table 2, entry 20)

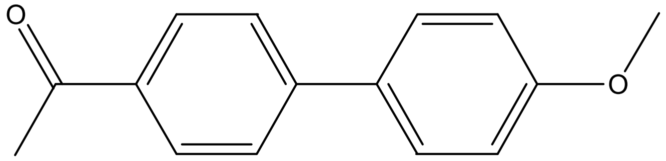

^{1}H-NMR (600 MHz, $CDCl_3$) δ 8.00 (d, *J* = 8.3 Hz, 2H), 7.65 (d, *J* = 8.3 Hz, 2H), 7.58 (d, *J* = 8.6 Hz, 2H), 7.00 (d, *J* = 8.6 Hz, 2H), 3.85 (s, 3H), 2.63 (s, 3H).

4'-Chloro-4-methoxybiphenyl (Table 2, entry 21)

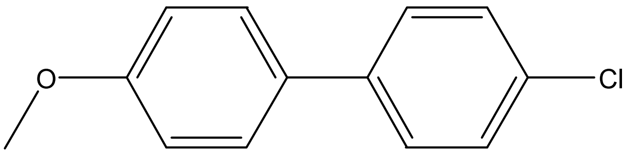

^{1}H-NMR (600 MHz, $CDCl_3$) δ 7.49–7.46 (m, 4H), 7.38 (d, *J* = 8.4 Hz, 2H), 6.97 (d, *J* = 9.2 Hz, 2H), 3.84 (s, 3H).

4-methoxybiphenyl (Table 2, entry 22-24)

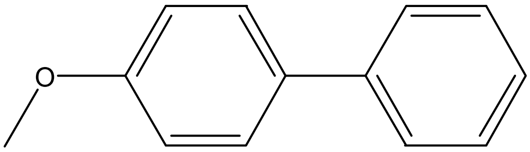

^{1}H-NMR (600 MHz, $CDCl_3$) δ 7.54 (dd, *J* = 12.6, 8.3 Hz, 4H), 7.42 (t, *J* = 7.6 Hz, 2H), 7.33–7.24 (m, 1H), 6.99 (t, *J* = 5.8 Hz, 2H), 3.85 (s, 3H).

4'-Methoxy-2-biphenylcarboxylic acid (Table 2, entry 25)

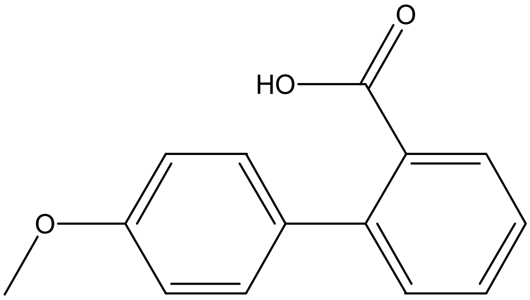

^{1}H-NMR (600 MHz, $CDCl_3$)δ 12.09 (s, 1H), 7.80–7.72 (m, 4H), 7.55–7.49 (m, 2H), 7.08–7.00(m, 2H). 3.80 (s, 3H).

4-Methoxy-4'-nitrobiphenyl (Table 2, entry 26)

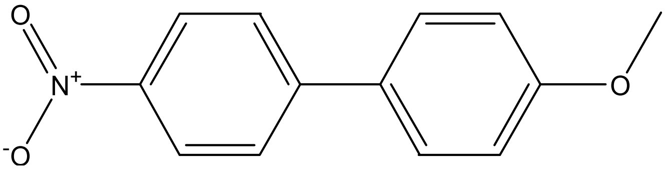

^{1}H-NMR (600 MHz, $CDCl_3$) δ 8.28 (d, *J* = 9.0 Hz, 2H), 7.69 (d, *J* = 9.0 Hz, 2H), 7.67(d, *J* = 8.4 Hz, 2H), 7.57 (d, *J* = 8.4 Hz, 2H), 7.00 (d, *J* = 9.0 Hz, 2H), 3.86 (s, 3H).
